# Using confocal microscopy and image processing to measure properties of sub‐resolution cell structures: Application to cardiac transverse tubules

**DOI:** 10.1111/jmi.70120

**Published:** 2026-05-19

**Authors:** Mark B. Cannell, Cherrie H. T. Kong

**Affiliations:** ^1^ Faculty of Health and Life Sciences Bristol Medical School University of Bristol Bristol UK

**Keywords:** cardiac myocytes, computer modelling, confocal microscopy, fluorescence microscopy, image processing, t‐tubules

## Abstract

Confocal microscopes increase the resolution of images by rejecting unwanted data from the out of focus regions of the specimen. This incurs a penalty in the recorded signal to noise ratio that may be partially ameliorated by inputting additional information during image processing. This is commonly seen in digital deconvolution techniques, where the point spread function (PSF) of the microscope is used to refine the image. By accounting for the interaction between the microscope PSF and labelled objects, information about sub‐resolution structures can be quantified. In the case of cardiac t‐tubules, we show the detailed 3D organisation and estimates of the t‐tubule diameter and approximately 50% of t‐tubules are below the classical optical resolution limit. By obtaining local t‐tubule volume (*V*) and surface area (*SA*) by using dual labelling, together with control of the PSF, a simple relationship between t‐tubule radius and the *V*:*SA* ratio can be derived. Solute movement can also be analysed by accounting for the shape of the illumination field during fluorescence recovery after photobleaching (FRAP). Using these methods, we show that the properties of murine and lagomorph ventricular t‐tubules are quite different.

## 1 INTRODUCTION

The development of the confocal microscope increased image contrast and resolution by rejecting the out of focus light present in widefield images (c.f. Figure 1A and B).^1^ For fluorescence imaging, the monochromatic light used to efficiently excite fluorochrome(s) can be provided by a laser. The use of a laser also avoids the need for a confocal excitation pinhole because the highly collimated laser light is focused to a diffraction limited spot in the focal plane.^2^, ^3^ The emission pinhole rejects out of focus light, but most confocal microscopes are not used at pinhole diameters that maximise resolution. This is because the resulting loss of in focus signal with small diameter pinholes increases photon noise and reduces image contrast (and hence resolution). The ‘sweet spot’ that gives the best overall image in terms of signal to noise and resolution occurs when then pinhole is set to about the diameter of the Airy disk (as defined by the distance to the first minima of the pupil diffraction pattern).^4^ Decreasing the pinhole diameter further only slightly increases resolution while severely impacting the signal that gets through the confocal pinhole.^4^ The performance of the confocal microscope is critically dependent on the aberrations in the system that alter the size and shape of the Airy disk^5^, ^6^ so care must be taken to make sure the pinhole(s) are correctly aligned with the source and spherical aberration is minimised by matching the refractive indices in the object space to the design of the objective. These issues are covered in greater detail in several reference texts.^5^, ^6^, ^7^

**FIGURE 1:**
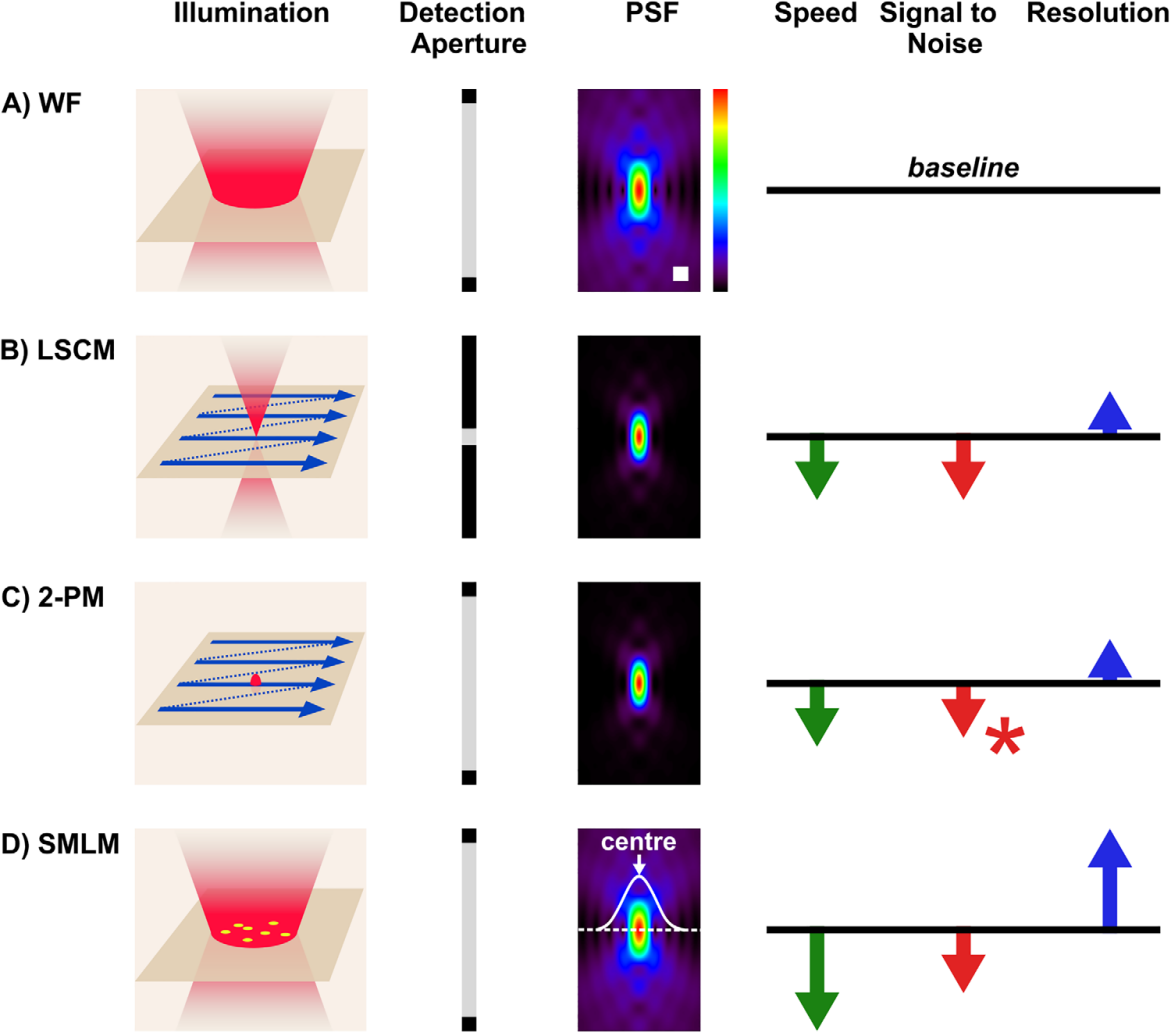
An illustrative overview of the balance between image qualities such as resolution, speed and signal to noise for various imaging modalities. (A) In widefield (WF), the entire field of view (FOV) is illuminated and the emitted light is detected within the objective aperture. The *x–z* image of the point spread function (PSF) shows that out of focus light is present in the image plane. The scale bar for the calculated PSFs shows 25 nm and the lookup table is shown. (B) Laser‐scanning confocal microscopy (LSCM) moves a laser spot across the FOV and the emitted light passes through a pinhole that rejects the out of focus light. The resulting PSF shows reduced power outside of the Airy disk. The scanning reduces the speed of image acquisition (green arrow) compared to in WF while the pinhole reduces signal to noise (red arrow) but improves resolution predominantly in *z* (blue arrow). (C) In 2‐photon microscopy (2‐PM), illumination is limited to a spot in the focal plane and detection is (or should be) unobstructed. The asterisk indicates that the practical resolution of 2‐PM is similar to LSCM, but reduced scattering at depth and reduced out of focus bleaching may improve 3D image signal to noise compared to confocal *z*‐stacks, so that the overall signal to noise ratio may be better. See text for detail. (D) Despite WF illumination, single molecule localisation microscopy (SMLM) protocols favour detection of one fluorescent molecule (indicated by yellow dots) at a time. This enhances resolution because the centre (marked by arrow) of the Airy disk defines the position of the molecule but sacrifices speed and signal to noise.

A variant of the confocal sectioning method is 2‐photon microscopy (2‐PM, Figure 1C), where the light source is an ultra‐fast (mode locked) laser that produces femtosecond pulses.^8^ The complexity of the mode locked laser light source was simplified by the development of ultra‐fast solid state lasers^9^ and the 2‐photon excitation completely removes the need for a confocal pinhole because excitation only occurs at the focal spot. The lack of a pinhole can greatly increase imaging depth in scattering samples—an effect that is further enhanced by the reduced Rayleigh/Mei scattering associated with the near infra‐red excitation wavelengths (see figure 4 in Ref. 10). The effect of spherical aberration is also reduced by lack of the pinhole and manifests mainly as a reduction in fluorochrome excitation rate. However, we have found that, in general, 2‐photon excitation cannot achieve the same excitation rate as conventional one photon excitation without significant in‐plane bleaching and even physical specimen damage. Nevertheless, if very large numbers of sections are needed for 3D reconstruction, this limitation is offset by the bleaching being limited to the current focal plane (see below).^11^ The excitation and bleaching rate with 2‐photon excitation is highly fluorochrome dependent as reflected in the ‘2‐photon cross‐section’, which determines the probability of 2‐photon excitation at a given wavelength,^12^, ^13^ as well as the pulse duration. Nevertheless, practical tests and theoretical considerations have shown that the resolution of a 2‐photon microscope is about the same as a regular confocal microscope,^4^, ^9^ unless the signal to noise becomes resolution limiting at depth in scattering samples.

The connection between signal to noise and resolution is manifest in the Shannon criterion^14^ which shows how resolution can be limited by the signal to noise ratio.^15^ This connection explains why increasing resolution in conventional microscope systems is problematic because a twofold increase in resolution of the sample object would be associated with an eightfold decrease in signal strength and a consequent $\sqrt{8}$ decrease in signal to noise (note that the detected voxel volume is proportional to the product of all 3 linear dimensions (*x*·*y*·*z*) while the signal to noise is proportional to the square root of the number of collected photons). While this limitation can be overcome by single molecule (Figure 1D) and structured illumination techniques (albeit at the expense of the imaging speed to acquire more photons and/or samples of the object in accord with the Lukosz invariance theorem^16^), detailed description of such ‘super‐resolution’ techniques are beyond the scope of this review (for further details, see Refs. 17, 18).

If resolution is limited by the signal to noise ratio, then deconvolution can improve image quality^19^, ^20^, ^21^, ^22^ (Figure 2A). If the point spread function^23^ (PSF, see Figure 1) is known, information is being added into the imaging pathway (which includes the image processing used to produce the final image) and this explains why resolution (and not just signal to noise) can be increased. For example, at the limit, the knowledge that the image arises from a single molecule can be used to provide a great increase (up to 10×) in resolution as seen in many of the current single molecule localisation methods (Figure 1D). A single fluorophore assumption also underlies super‐resolution radial fluctuations (SRRF) microscopy, where the likelihood of a fluorophore being present in a sub‐pixel is calculated and positions summed over time to improve resolution.^24^ Similarly, super‐resolution optical fluctuation imaging (SOFI) uses intensity fluctuations over time to help define fluorophore positions.^25^ However improved resolution comes at the expense of speed as described above.

**FIGURE 2:**
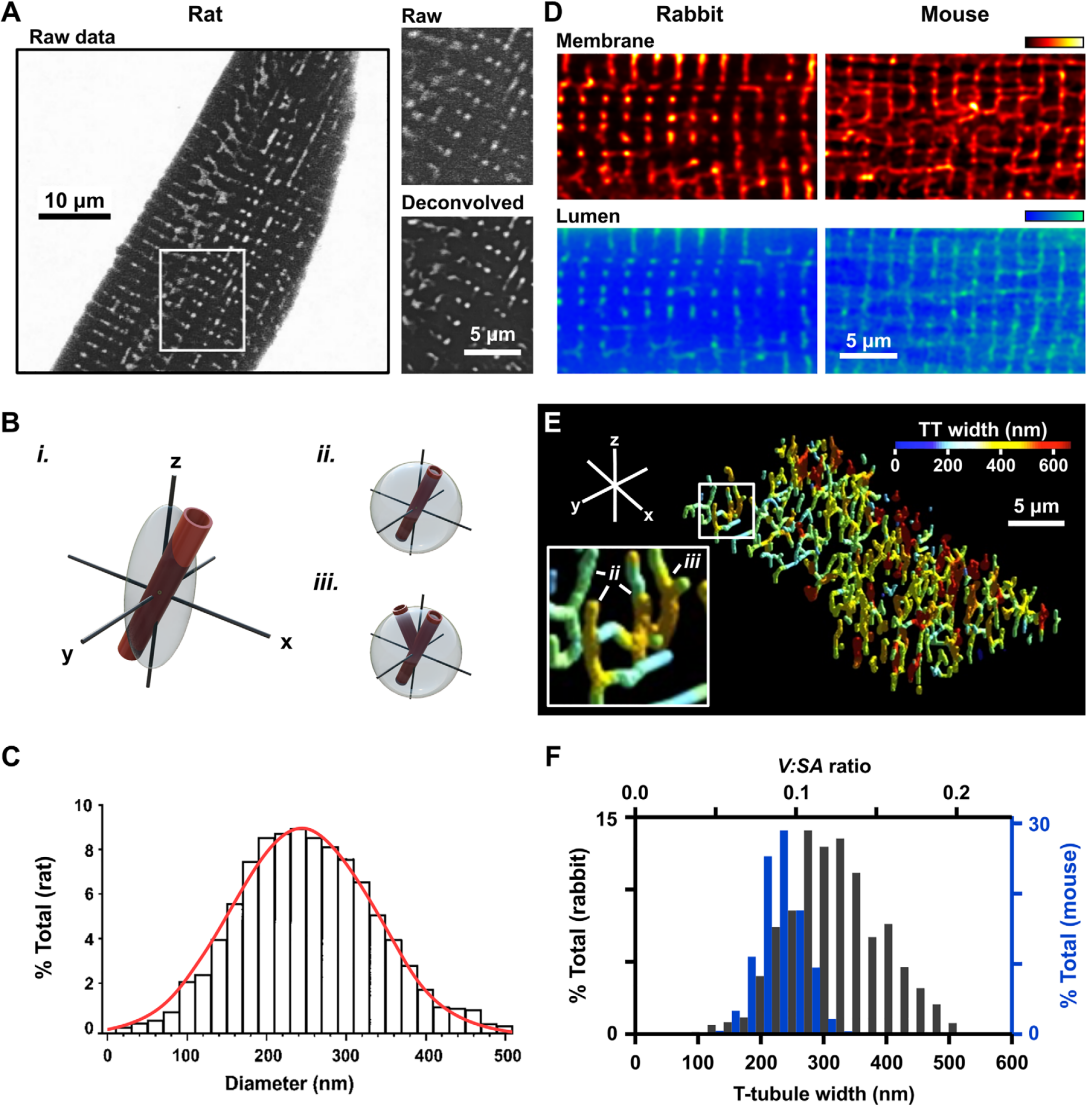
Estimating sub‐resolution t‐tubule widths in living cardiac myocytes. (A) Isolated rat ventricular myocyte superfused with 70 kDa dextran‐linked fluorescein. Insets show enlarged region before (top panel) and after (bottom panel) noise reduction by deconvolution. (B) Illustrates how dye signal from t‐tubules is sensitive to orientation within an asymmetric PSF **(i)**, but orientation does not matter within a spherical PSF **(ii)**. However, branching patterns **(iii)** need to be accounted for and this can be achieved by skeletonising the image. Exemplar branching patterns in t‐tubules are shown in the inset of panel E. (C) Rat t‐tubule diameters obtained by image processing (see text). T‐tubule diameters had an approximately Gaussian distribution (red line), with an average of ∼250 nm (range 50–500 nm), so that >50% of tubules were below the resolution limit of the confocal microscope. (D) Deconvolved images of ventricular t‐tubule labelling in rabbit (left panels) and mouse (right panels) myocytes stained with the membrane dye, FM4‐64, and extracellular space (t‐tubule lumen) labelled with calcein. Lookup tables are shown at right. (E) Ccalculated t‐tubule widths in mouse mapped onto the corresponding network skeleton (dilated 3× for display purposes). The t‐tubule diameter colour lookup table is shown at right with the optical axes shown at left. Inset shows the region indicated by white box at 3× magnification. **(ii)** t‐tubules that are nearly parallel to the optical axis (see *Bii*) and **(iii)** a branchpoint (see *Biii*). (F) The *V:SA* ratio and calibrated t‐tubule diameter (assuming near cylindrical geometry) for a rabbit (grey bars) and a mouse (blue bars) myocyte. Data in A–C and D–F adapted from Refs. (19) and (36), respectively.

In this short review, we describe some techniques we have used to increase informational content of image data. Initially we used constrained iterative Richardson‐Lucy deconvolution with a known PSF to improve signal to noise in images of transverse (t‐) tubules inside living cardiac ventricular myocytes.^21^ This image data is then processed via PSF modification and skeletonisation to provide a measure of t‐tubule diameter down to a limit of ∼50 nm across the entire cell network. A similar image processing strategy is shown to provide a measure of the local surface area to volume ratio, which is important for the electrical and passive transport properties of t‐tubules. Finally, we show how fluorescence recovery after photobleaching (FRAP) combined with detailed modelling of geometry and diffusion provides new insight into solute exchange within t‐tubules.

## 2 USING INTENSITY INFORMATION TO EXCEED THE NORMAL DIFFRACTION LIMIT

The signal from a fluorophore depends on the product of local concentration and local volume in the imaging plane, as well as convolution by the microscope PSF. In most applications, it is the local concentration that gives information about the location and abundance of the fluorescent molecule(s) (e.g. in fluorescence immunocytochemistry); however, the dependence of signal intensity on local volume can be exploited. The basic geometric properties of the cardiac t‐tubular system is critical for passive propagation of the electrical signal^26^, ^27^, ^28^ into the interior of the myocyte as well as solute exchange (for review, see Ref. 26, 29). While recent optogenetic depolarisation of murine t‐tubules has shown that 10%–15% may be intrinsically electrically excitable,^30^ the electrical load imposed by non‐excitable tubules may still compromise depolarisation in t‐tubule altering disease states^31^, ^32^, ^33^ (and other species), so measures of t‐tubule geometry are important.

While some of the t‐system had been analysed by electron microscopy (EM) and reconstruction from thin sections,^34^, ^35^, ^36^, ^37^ light microscopy can give information over a larger field of view and number of samples. Since the t‐system can be filled with a fluorescent dye of measurable concentration (Figure 2A and D; see also Ref. 38), the local t‐system fluorescence signal, *I_t_*, should be related to the local t‐tubule volume and the interaction between the local geometry and the microscope PSF:

$$I_{t}={∰}_{D}\mathrm{PSF}\left(x,y,z\right)⊗t\left(x,y,z\right)\mathrm{dx}\;\mathrm{dy}\;\mathrm{dz}:D\;\subset \mathrm{imag}\mathrm{ing}\;\mathrm{volu}\mathrm{me}.$$

The *t*(*x*,*y*,*z*) term depends on the local dye concentration, the local t‐tubule radius, angle to the optical axis (Figure 2Bi–ii), as well as the branching pattern within the local volume (Figure 2Biii) (⊗ is the convolution).^19^ The dependence of the t‐tubule signal on orientation arises from the asymmetry of the confocal (or 2‐photon) PSF (Figure 2Bi) because t‐tubules that are parallel to the optical axis will produce more signal than those orthogonal to the axis (all other terms being equal). This problem is overcome if the *effective* PSF is made spherical (Figure 2Bii), which is achieved by convolving the 3D image stack with a 3D Gaussian function whose *x*,*y* dimension is equal to the *z* resolution of the microscope PSF and *z* dimension equal to the microscope *x*,*y* resolution (i.e. a spherical PSF). This now blurred image sacrifices some *x*,*y* resolution in exchange for an improved signal to noise ratio in the dye signal. The loss of *x*,*y* resolution is inconsequential because t‐tubule location is provided by skeletonisation^39^, ^40^ (see below) of deconvolved high resolution images. It is worth noting that the *accuracy* of the t‐tubule centroid is improved by skeletonisation, similar to the way in which the location of the centroid of the Airy disk is used to define localisation in single molecule super‐resolution techniques.^17^, ^18^

The local intensity of fluorophore signal is also affected by branching and curvature within the PSF resolution (Figure 2Biii; see also figure 2D and E in Ref. 19). This problem is also solved by skeletonisation of the t‐tubule image stack. After convolving the skeletonised t‐tubules with the same spherical PSF, a reference image is obtained where all t‐tubules have the dimensions of a voxel, but the signal depends on the branching pattern. Therefore, division of the modified t‐tubule image by this reference image gives a new image proportional to the local (cylindrical) t‐tubule volume and dye concentration (see figure 2 in Ref. 19). The constant of proportionality is provided by the signal from outside the cell where both the dye concentration and the imaging volume (the PSF) are known. It is worth pointing out that the spherisation and skeletonisation steps in this method are readily achieved in most 3D image processing packages (e.g. ImageJ/FIJI^41^). An example processing pipeline (MATLAB 2023b) is available at https://github.com/cherrieKong/ttubule_analysis.

This novel image processing strategy^19^ yielded some unexpected results for rat ventricular t‐tubules: (1) the t‐tubule diameters were more variable than expected, ranging from <50 up to 500 nm (Figure 2C); (2) the t‐tubule system was more extensive than expected from EM thin sections; (3) it is possible to quantify a complex cell structure whose average size is less than the wavelength of light and the resolving power of the microscope. It should be noted that in order to use this strategy, individual structural elements must be optically separable. If this is not the case, the physical measure would reflect the summation over all the sub‐resolution objects within the focal volume. STED^42^ imaging also suggested average t‐tubule diameters consistent with our histograms, but did not detect t‐tubules <100 nm in diameter.^43^ Nevertheless, the resolution of the lumen in such t‐tubules is remarkable,^43^ although the lack of smaller tubules (as detected by our method) may be due to a lower signal to noise ratio in live cell STED.

While the above analysis could be used give insight into the solute volume within the t‐system and changes in t‐tubule structure,^32^, ^44^, ^45^ it does not address the question of the local surface area to volume ratio, which is important for understanding bulk diffusive and transmembrane transport. To address this problem, we extended the method to include two dyes:^46^ one giving a signal from the local solute volume inside the t‐tubule (*S_VT_*) and the other the surface membrane area of the t‐tubule (*S_AT_*) (Figure 2D). Again, making the PSF spherical removed the orientation component of the signal so that for circular cross‐section t‐tubules:

$$\;\frac{S_{VT}}{S_{AT}}=\frac{C_{Vol}}{C_{Area}}\;\cdot \frac{r}{2},$$

where *C_Vol_* and *C_Area_* are calibration constants relating to the concentration of fluorophores in the bathing solution and membrane. For markedly elliptical cross‐sections, the relevant equation is:

$$\;\frac{S_{VT}}{S_{AT}}=\frac{C_{Vol}}{C_{Area}}\cdot \frac{AE}{\left(1+E\right)\left(1+\frac{3h}{10+\sqrt{4-3h}}\right)};\;h={\left(\frac{\left(1-E\right)}{\left(1+E\right)}\right)}^{2},$$

where *E* is the ellipticity as defined by the ratio of minor to major elliptical axes, and *A* is the semi‐major TT dimension. However, the effect of ellipticity is small (∼10%) for both mouse and rabbit t‐tubules with *E* ∼0.75 (as determined by electron tomography,^46^ but see Ref. (43) for *E* ∼1.0 in mouse t‐tubules imaged using STED).

To obtain *C_Vol_*/*C_Area_*, it should be noted that, when the microscope focus is at the outer surface of the cell, the volume signal being measured (*S_VS_*) should be half of that recorded when the microscope focus is in the bath because the PSF is bisected by the cell surface membrane. Similarly, the surface signal *S_AS_* arises from a plane bisecting the PSF. For a Gaussian PSF:


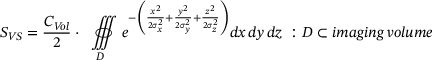

and

$$S_{\mathrm{AS}}=C_{\mathrm{Area}}\;\cdot {∰}_{D}e^{-\left(\frac{y^{2}}{2\sigma_{y}^{2}}+\frac{z^{2}}{2\sigma_{z}^{2}}\right)}\mathrm{dy}\;\mathrm{dz}\;:D\subset \mathrm{imag}\mathrm{ed}\;\mathrm{cell}\;\mathrm{surf}\mathrm{ace}.$$

After modifying the effective system PSF with Gaussian blurring to become symmetrical (and therefore precisely known), dividing the above equations and integrating the result gives:

$$\;\frac{S_{VS}}{S_{AS}}=\frac{C_{Vol}}{C_{Area}}\;\cdot \frac{FWHM}{4}\cdot \sqrt{\frac{\pi }{\ln2}},$$

where *FWHM* is the full width at half maximum of the spherical Gaussian PSF. The ratio of the normalised volume signal (*V*) to that of the surface area (*SA*) in any sub‐resolution circular cross‐section t‐tubule is therefore:

$$V:SA=\frac{r}{FWHM}\;\cdot \sqrt{\frac{\ln2}{\pi }}.$$

See Ref. (46) supplement for further detail. This simple formula can be used to not only analyse a cylindrical t‐tubule system but could be applied to (for example) labelled neurons to examine the space constant of dendrites and non‐myelinated axons, provided the individual neuronal structures are separable. Separation of closely spaced cellular structures may be achieved by selective labelling, or in the case of whole cells, single cell injection of the fluorescent marker during patch‐clamp experiments (e.g. see figure 25.9 in Ref. 21). Furthermore, this approach can be generalised to examine almost any sub‐resolution structure provided the basic geometry is known.

This method has been used to analyse t‐tubules in mouse and rabbit ventricular myocytes (Figure 2D). Figure 2E shows an exemplar illustration of the network‐wide t‐tubule dimension estimates and the local variability is apparent. Quantitative analysis revealed quite different distributions of t‐tubule diameters between the two species (Figure 2F). Mouse cardiac t‐tubule diameters were generally less than the diffraction limit of the confocal microscope, and were significantly smaller than seen in rabbit (0.169 ± 0.015 vs. 0.356 ± 0.018 µm, respectively, *p* < 0.001). Since some rabbit t‐tubules were above the diffraction limit, it was possible to check the veracity of the *V*:*SA* method in the same t‐tubule, and this showed that the *V*:*SA* method was in general agreement with direct measurement (see figure 2 in Ref. 46).

## 3 USING CONFOCAL MICROSCOPY TO MEASURE DYNAMIC SOLUTE CHANGES

T‐tubule structure should affect exchange of solutes with the possibility of ionic accumulation/depletion during electrical activity deeper within the cell.^47^ Rapid solution changes of the cell superfusate can be used to probe how solutes pass from the bulk extracellular space to the t‐tubule lumen at the interior of the cell;^48^, ^49^, ^50^ however, computational analysis showed that unstirred layers around the cell affect the ability to rapidly change solutions at the cell surface (see Figure 2 in Ref. 51). Fluorescence recovery after photobleaching (FRAP)^28^, ^52^, ^53^ could be used to avoid unstirred layers; however, the shape of the microscope illumination beam used for photobleaching (Figure 3A) adds complexity to data interpretation. This complexity arises because, even for in focus in‐plane illumination, the illumination pattern is not a uniform disk.^54^, ^55^ While 2‐photon excitation^10^ can reduce the photobleaching geometry to a single plane (Figure 3A, right panel), the thinness of the photobleached region (∼1 µm) would result in a very fast (ms) fluorescence recovery in 3D objects that would be hard to measure in most confocal microscopes. Furthermore, while an assumption of a uniform solute diffusion coefficient may be necessary to enable interpretation of FRAP time course in terms of solute movement in parallel, uniform cylinders,^28^ the structure of the t‐system is very anisotropic (see above). Fluorescence correlation spectroscopy (FCS) and related techniques can also probe microscopic solute diffusion coefficients, but interpretation of the FCS signal hinges upon a number of factors, which may be problematic^56^ within a complex t‐tubule system (e.g. volumes smaller than the PSF, very low dye concentrations, tortuosity factors, etc.). Moreover, it is unclear whether FCS would be a better tool to measure macroscopic solute exchange between the cell interior and exterior.

**FIGURE 3:**
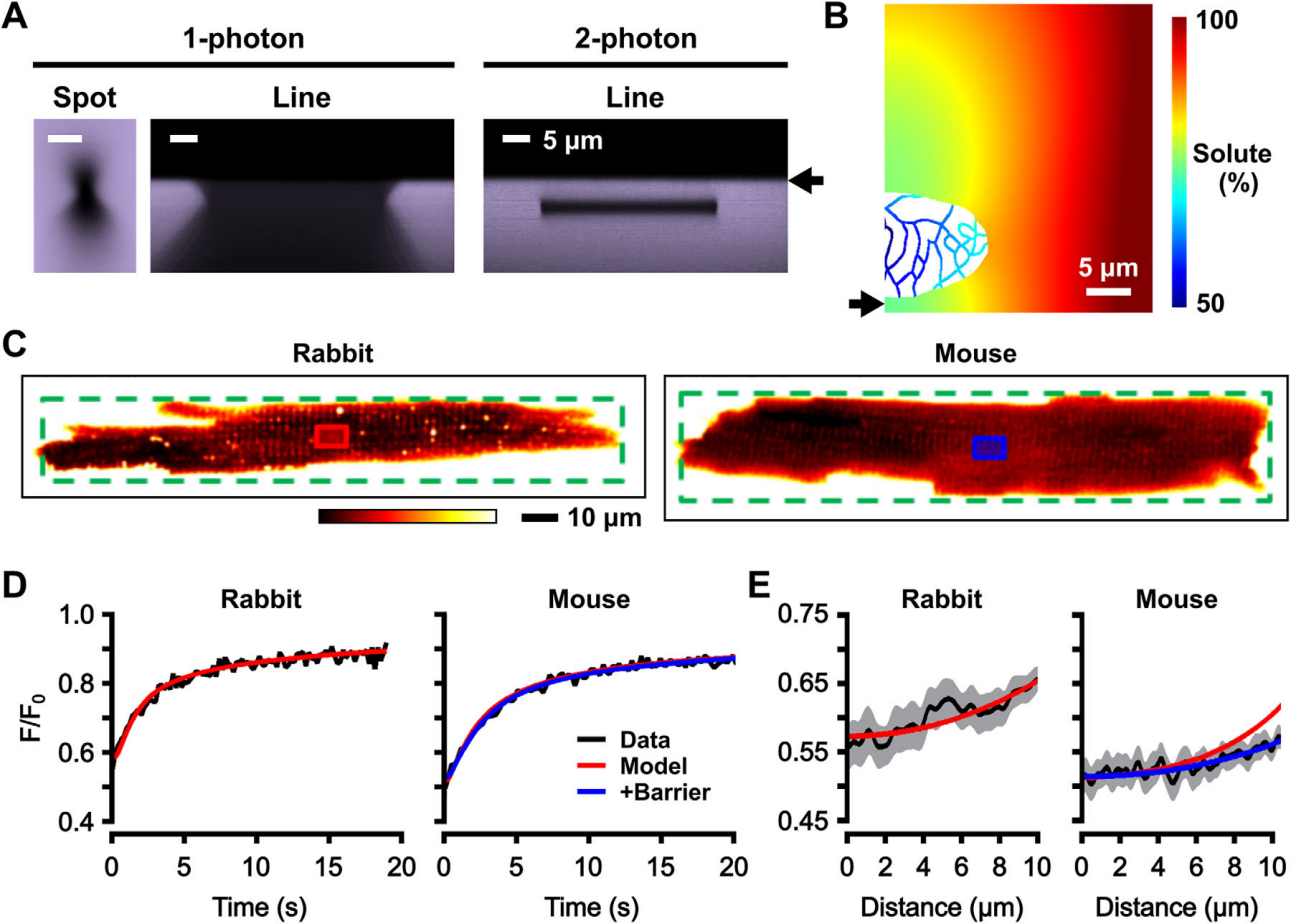
Quantification of solute movement in t‐tubules using fluorescence recovery after photobleaching (FRAP) and confocal microscopy. (A) *x–z* images of photobleaching profiles during (from left to right): 1‐photon excitation at a fixed spot, 1‐photon excitation along a line, or 2‐photon excitation along a line. The black arrow indicates the coverslip edge. Note the difference between bleaching patterns depending on imaging modality. (B) Photobleaching beam geometry affects the apparent rate of diffusion within t‐tubules. Image shows the calculated concentration of unbleached solute at the end of photobleaching. Note that solute above and below the cell (as well as within the t‐tubules) is bleached non‐uniformly (the time‐course of bleaching distribution is shown in as a movie attached to Ref. 51). (C) Rabbit (left panel) and mouse (right panel) ventricular myocytes incubated in a ∼1 kDa solute. The green dashed boxes show the photobleaching areas, and the smaller boxes indicate the region that was recorded for FRAP. The colour bar shows the lookup table used for both images, spatial scale at bottom. (D) The corresponding FRAP time course (black lines) and model fits (coloured lines). (E) The radial fluorescence profiles at the end of photobleaching. In D and E, rabbit FRAP time and spatial profiles could be fitted using a single diffusion coefficient (red lines), while for mouse, the model fit required an additional surface barrier to diffusion (blue lines). Photobleaching profiles during repeated imaging were adapted from Ref. (11) and all other data from Ref. (51).

Since the photobleaching cone can be measured (or computed), this was used as a boundary condition in a computer model that simulated FRAP within t‐tubules (Figure 3B). Typical cell and FRAP time courses (with model fits) are shown in Figure 3C and D, respectively. While the rabbit FRAP data was consistent with a single diffusion coefficient across the cell, this was not the case for mouse where the spatial data could not be fitted without including a surface barrier to diffusion (Figure 3E). Subsequent EM tomography showed that there appeared to be a more complex entrance to the wider t‐tubule network in mouse.^51^, ^57^ This approach also revealed that on average, the apparent rate of diffusion within rabbit and mouse t‐tubules was ∼52 and ∼23 µm^2^/s, respectively, which is ∼5‐ to 12‐fold slower than free diffusion but faster than the 22‐ to 35‐fold reduction in free diffusion reported previously for mouse and rat cardiac myocytes.^28^, ^58^ This reduction in apparent rate of diffusion for both species could be entirely explained (within experimental variability) by analysis of t‐tubule geometry (see above) and quantification of the effect of tortuosity, presence of longitudinal t‐tubules, varicosities and surface barrier (see figure 7 in Ref. 51).

## 4 SUMMARY

We have shown that detailed consideration of the imaging process can be used to increase the amount of information that can be obtained from confocal and 2‐PM images. Light microscopy has the particular advantage that large areas and/or number of cells can be analysed without the need to fix samples so that derived morphology may more closely reflect the living cell. Since resolution and/or signal to noise can be enhanced by supplying information into the image processing pathway, the diffraction limit can be overcome. We have used this approach to obtain best estimates of t‐tubule structure in cardiac myocytes and how these parameters impact solute exchange. We also showed that detailed analysis of FRAP illumination patterns and data is key for clarifying macroscopic solute exchange times. While this review focuses on our cardiac t‐tubule studies using 2‐PM and confocal microscopy, the approaches we have developed may be applicable and/or extendable to other cellular structures and problems in biophysics. More recent advances that can further improve the basic resolution of the confocal microscope (e.g. SRRF^24^ or Airyscan^59^) can also be combined with our methods to extract physical properties of small cellular structures. Since the analysis of geometry using system PSF modification (and skeletonisation) can be readily achieved using open‐source software packages, our approach could be used for quantification of other thin structures such as those associated with neuronal processes (assuming that suitable markers are available and can be introduced to the selected cells).
